# Is Early Life Adversity Associated With Adult Stress in a Wild Rodent?

**DOI:** 10.1002/ece3.71065

**Published:** 2025-03-27

**Authors:** Alyssa Y. Kong, Xochitl Ortiz‐Ross, Daniel T. Blumstein

**Affiliations:** ^1^ Department of Ecology and Evolutionary Biology University of California Los Angeles California USA; ^2^ The Rocky Mountain Biological Laboratory Crested Butte Colorado USA

**Keywords:** cumulative adversity index, cumulative stress, early life effects, glucocorticoids, multiple stressors, wild rodent

## Abstract

The period before sexual maturity is a sensitive life stage where most development and change occur. Studies in humans and other animals show that early adverse experiences contribute to poor health and survival. However, the mechanisms are still unclear. Some have found that early life adversity (ELA) can lead to elevated glucocorticoids later in life, dysregulate the stress response, and increase the impact of later stressors. However, most animal studies have focused on individual stressors. Protecting wild populations that are exposed to multiple stressors requires a better understanding of the physiological consequences of several co‐occurring stressors. We used a cumulative adversity index (CAI) to ask whether early adverse experiences were associated with increased levels of adult fecal glucocorticoid metabolites (FGM) in wild female yellow‐bellied marmots (*Marmota flaviventer*). We found a significant interaction between adversity and elevation that explained variation in FGMs. Thus, we infer that ELA can modulate FGMs, but contrary to similar research in other mammals, the trend was toward downregulation under more environmentally relaxed conditions (lower elevation). Our results highlight the value of studying the relative importance of early and later stressors in the physiology of different wild taxa when investigating the mechanisms of early life adversity.

## Graphical abstract

Using a cumulative adversity index, we found that adult glucocorticoid levels in wild yellow‐bellied marmots vary with the number of early adverse experiences, but their relationship is dependent on elevation. Under more relaxed environmental conditions, at lower elevations, glucocorticoids decrease with increasing adversities, while there is a slight opposite trend at higher elevations, where environmental conditions tend to be harsher.

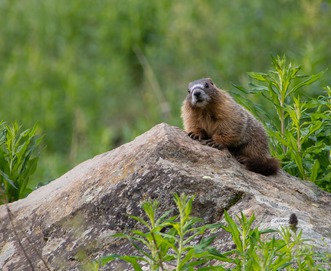

## 1 Introduction

Early life is defined as the period between conception and sexual maturity (Henry and Ulijaszek 1996). It is a sensitive stage of life because substantial development, growth, and biological change occur during this period (Knudsen 2004). Experiences in early life form the foundation for individual behavior (McEwen 2003), neural network structure (Singer 1995), and health that will last their lifetime (Elwenspoek et al. 2017). Therefore, facing adversity during this important period can alter biological pathways and potentially lead to chronic health problems (Nelson and Gabard‐Durnam 2020). For example, in humans, children who received maltreatment (defined as abuse or neglect) or experienced trauma (such as loss of a parent) were at higher risk for all common mental disorders and poorer health later in life (Green et al. 2010). Similarly, in wild female baboons ( *Papio cynocephalus* ) those who experienced higher cumulative early‐life adversity (ELA) were more socially isolated and had shorter lifespans (Tung et al. 2016).

However, the mechanism(s) by which ELA reduces survival and fitness are not well understood. A commonly proposed hypothesis suggests that ELA reduces adult fitness by increasing the synthesis of adrenal (or “stress”) hormones such as glucocorticoids (GCs), (Evans and Kim 2007; Hammen et al. 2000; McLaughlin et al. 2010)—a protective homeostatic response to stressors (McEwen 1998). GCs are a metabolic hormone that helps mediate energetic demands; they divert and mobilize energy where needed to maintain regular body function. As such, when energy demands increase in response to environmental challenges, GC levels are expected to increase. This is how GCs, which are easily measured, have come to be used as proxies for environmental stress (Beehner and Bergman 2017), including ELA. Indeed, studies have found a positive association between ELA and high levels of GCs in humans (Evans and Kim 2007) and baboons (Patterson et al. 2021; Rosenbaum et al. 2020).

While GCs are an adaptive, protective response to an immediate challenge, there is also evidence, primarily in humans and laboratory animals, that overexposure to such hormones (or “chronic stress”) can lead to decreased health and fitness (Epel et al. 2004; Homyack 2010; McEwen 1993; Sapolsky et al. 2000). A strong association between GCs and survival was also found in wild female baboons ( *Papio cynocephalus* , with some admixture from a closely related species, the olive baboon, *Papio anubis* ; Campos et al. 2021). Such overexposure is also known as “allostatic load” (McEwen 1993) or “homeostatic overload” (Romero et al. 2009).

A strong biomedical literature bias on the association between GCs and fitness has led many to begin interpreting high GCs not only as a measure of environmental stress, but also as a measure of poor fitness (Bonier et al. 2009). However, few studies have supported this association in wild animal populations (Boonstra 2013).

Additionally, high GCs are not the only evidence of chronic or cumulative stress. Repeated activation of the hypothalamic–pituitary–adrenal (HPA) axis (also referred to as the “stress response”) can also lead to a dysregulated response to stressors, meaning that the response is no longer appropriate and can lead to decreased health and fitness (Sapolsky et al. 2000). ELA in humans has been associated with such dysregulation, resulting in either sensitization (increased reactivity) or habituation (decreased reactivity). Both can increase vulnerability to stressors later in life. Therefore, individuals with greater ELA could have more difficulty coping with stressors and, consequently, may experience greater fitness consequences later in life (McEwen and Seeman 1999). Importantly, such dysregulation can be associated with decreased rather than increased baseline GCs.

Despite extensive research on ELA in humans, investigating long‐term fitness consequences is challenging, and the use of longitudinal data is rare. Nonhuman systems offer an opportunity to study the long‐term impact of ELA across multiple generations and to discover the mechanisms and life history changes associated with heightened adult morbidity and mortality. However, cumulative ELA and multiple‐stressor studies on free‐living wildlife are still very limited, especially for terrestrial animals (Orr et al. 2020). Studies in natural populations have focused mainly on the responses to single early life stressors (such as food scarcity) (Blumstein et al. 2016; Cordes et al. 2020; Descamps et al. 2008; Krause and Liesenjohann 2012; Ozgul et al. 2010; Van Vuren 2001). However, natural populations experience a combination of early stressors that can have a cumulative impact on an individual's fitness (Ortiz‐Ross and Blumstein 2024; Tung et al. 2016). Such studies are challenging because they require extensive longitudinal data across multiple generations. Furthermore, the analyses required to include numerous stressors can become complex. To address this need, we leveraged six decades of longitudinal research on a wild population of yellow‐bellied marmots (*Marmota flaviventer*) near Gothic, Colorado, USA, and used nearly two decades of data to conduct an analysis of the effect of cumulative ELA on fecal glucocorticoid metabolites (FGMs; a measure of GCs) in adults.

Yellow‐bellied marmots are large ground squirrels that hibernate 7–8 months a year and live along an elevational gradient. The harsh environmental conditions they experience, their long hibernations, and the short time frame they have available to accumulate resources make this an interesting species with which to investigate adversity. Furthermore, because early‐life for marmots is only 2 years, much of their early life environment has been recorded (individuals are closely observed from their first emergence as pups until they disperse or die). This data set provides a unique opportunity to address the long‐term impact of ELA on adult stress. Within this population of yellow‐bellied marmots, increases in ELA have been shown to drastically reduce pup survival and female adult longevity (Ortiz‐Ross and Blumstein 2024). Furthermore, several detrimental fitness consequences have been reported in association with high FGMs, including a higher risk of mortality during hibernation (Wey et al. 2015) and lower offspring survival (Pinho et al. 2019).

Here, we aim to determine whether adult FGM levels are associated with exposure to ELA and to gain better insight into the potential role of GCs in mediating the impact of ELA on longevity. More specifically, we investigated the association between cumulative ELA, measured by a recently developed cumulative adversity index (CAI) (Ortiz‐Ross and Blumstein 2024), and FGM levels in adult female yellow‐bellied marmots. Adapted from the human literature (Rutter 1983), the CAI represents the sum of all adversities experienced in early life (e.g., high predation risk or maternal loss). Here, an adversity was defined based on its known impact on any fitness aspect or correlate in this population (see methods). Given our understanding of ELA in humans and other species, we expected that adult (≥ 2 years old) female yellow‐bellied marmots that experienced higher ELA would also exhibit atypical FGM levels. Adult marmots could exhibit either elevated FGMs (potentially indicating sensitization) or lower FGMs (potentially indicating habituation). Either would indicate a possible dysregulation of the stress response. On the other hand, a lack of association would indicate that varying FGMs may not be a mechanism by which ELA affects survival in marmots. Our results will give us a better understanding of the extent to which GCs represent a mechanism by which ELA affects adult survival, fitness, and health in a wild animal population that regularly experiences harsh and unpredictable environmental conditions while undergoing drastic seasonal changes in metabolism.

## 2 Materials and Methods

### 2.1 Study System and Data Collection

To determine whether ELA is associated with adult stress, we used longitudinal data collected from 2002 to 2020 from a population of yellow‐bellied marmots located around the Rocky Mountain Biological Laboratory (RMBL), in Gothic, Colorado. Spanning an elevational gradient of around 300 m, this population has been historically split into higher elevation colonies and lower elevation colonies. There is little to no migration between the two valleys, which experience considerably different environmental conditions. Most notably, higher elevation colonies tend to experience longer, harsher winters and shorter springs (Van Vuren and Armitage 1991). This also results in several differences in life history.

For the past 62 years, this marmot population has been observed during peak activity (0700 to 1100 h; 1600 to 1900 h) from mid‐April to mid‐September, their active season. Observational data provide detailed information on the life of individual marmots from when they first emerge from natal burrows as pups until they disperse or die. Therefore, we have accurate data on the environment each marmot was exposed to in its early‐life (from emergence around 30 days after birth to sexual maturity at age 2). Since males are the dispersing sex, most of our longitudinal data comes from females. We therefore chose to consider only females in this study, and our adversity measures reflect known negative impacts on female fitness. Males likely face a different set of adversities and were excluded.

In addition to near‐daily observations, marmots are live trapped biweekly in Tomahawk traps baited with Omalene 100 horse feed that are set at the entrances of their burrows. At capture, individuals are sexed and measured, their reproductive status is noted, and blood, fecal and hair samples are collected. Hair samples are used for subsequent microsatellite analysis to confirm paternity and, in the case of mixed litters, maternity (Blumstein et al. 2010). When first captured, all marmots received unique ear tags, and we used Nyanzol cattle dye to create a unique mark on their dorsal pelage. Feces are collected in plastic bags and immediately placed in coolers to be transferred to a −20°C freezer within 2 h. The samples are then transported at −20°C to UCLA for the extraction of FGMs (details in Blumstein et al. 2006), which have been validated as a proxy for physiological stress in this population (Smith et al. 2012). FGMs were then quantified using a corticosterone radioimmunoassay kit (RIA; MP Biomedicals, Costa Mesa, CA), which was validated for this population in Smith et al. (2012).

All field procedures were approved by a UCLA research protocol (2001–191, renewed annually) and under permits issued by the Colorado Division of Wildlife (TR‐519, renewed annually).

### 2.2 Cumulative Adversity Index

To quantify ELA, we used a cumulative adversity index (CAI), a previously validated measure that is closely associated with longevity in yellow‐bellied marmots (Ortiz‐Ross and Blumstein 2024). The index included measures of adversity known to affect various fitness components in female yellow‐bellied marmots. Once again, only females were considered in this study because this is where most of our knowledge lies, and the set of adversities may be different for males.

We computed a CAI by adding the nine adversity measures described below as binary measures (1 = adversity; 0 = no adversity) following (Ortiz‐Ross and Blumstein 2024). The cutoff for adversity was determined based on the upper quartile of the total distribution for each adversity measure; therefore, the experiences shared by 75% of the population were considered ‘the norm’ while the extreme experienced by only 25% of the population was considered adverse.

### 2.3 Adversity Measures

*Delayed onset of the growing season*, when vegetation emerges, is associated with poor body condition in marmots. The longer it takes for food to become available, the more marmots deplete their fat storage. Furthermore, later springs also result in a shorter season, which means marmots have less time to forage and gain weight before hibernating again. Both may be associated with a reduced probability of successfully breeding and a decreased chance of winter survival the following year (Blumstein et al. 2016; Cordes et al. 2020; Ozgul et al. 2010). We quantified the start of the growing season using NDVI satellite imagery data from the USGS eMODIS dataset (Jenkerson et al. 2010) which detects the timing and duration of green‐up. We used hand‐drawn polygons of our colony areas to obtain colony‐specific measures for each year from 2002 to 2020.

*Summer drought* is correlated with lower food abundance and marmot body mass gain, which is associated with a lower chance of survival from hibernation (Blumstein et al. 2016; Ozgul et al. 2010; Van Vuren 2001). We calculated total precipitation in the months of June, July, and August (in mm) using the “Global Summary of the Month” data set from the Crested Butte, CO weather station (freely provided by the National Oceanic and Atmospheric Administration, National Centers for Environmental Information).

*High predation pressure* is associated with high summer mortality in young pups (Van Vuren 2001). We calculated a predation index as the number of predators detected in a colony in April, May and June (by July, the vegetation prevents us from seeing many predators) divided by the time we spent watching the colony.

*Male‐biased litters* are associated with higher testosterone for females in the litter, which results in masculinized females. Such females tend to have lower survival in the first 2 years of life and lower reproductive success later in life (Monclús and Blumstein 2012). We calculated the sex ratio of litters at emergence from detailed observation and focused trapping of emerging litters.

*Large litters* are thought to increase competition among siblings. They are associated with a smaller weaning mass, which contributes to reduced survival, as more food must be shared (Armitage 2014a). We calculated litter size at the time of emergence from detailed observations and focused trapping of the emerged litters.

*Late weaning date* is associated with decreased survival; later emerging pups have less time to gain enough weight to survive hibernation (Ozgul et al. 2010). We calculated weaning date from the date the first individual in a litter was observed emerging from the burrow. In the case of mixed litters, it was not always possible to obtain an exact weaning date, so we were cautious in including animals when there were multiple litters emerging from the same burrow unless we were certain about their emergence dates.

*Low maternal mass* early in the season is associated with smaller offspring at emergence; heavier mothers can provide more resources, and their offspring are heavier when they emerge (Monclús et al. 2011). We estimated the female body mass on 1 June based on the Best Linear Unbiased Predictions (BLUPs) from a linear mixed effects model (details in Ozgul et al. 2010). This provides a consistent way to account for relative body condition since mass varies greatly throughout the season, and not all individuals are trapped the same day.

*High maternal FGMs* have been shown to contribute to reduced offspring survival through increased offspring FGMs (Pinho et al. 2019). Indeed, maternal FGMs can be directly transmitted to offspring during pregnancy or lactation (Monclús et al. 2011).

*Maternal loss* during early life has been shown to contribute to decreased survival and longevity of marmots (Ortiz‐Ross and Blumstein 2024). Previous work has shown that females are more likely to be recruited into the group if their mothers are still present and are more likely to disperse if their mothers are absent (Monclús et al. 2011). We noted whether individuals lost their mothers in their first or second year of life.

### 2.4 Data Analysis

To evaluate whether cumulative ELA is associated with adult physiological stress, we fitted a linear mixed effects model with FGM levels, log_10_ transformed, as our dependent variable and the CAI as our predictor variable. Other factors known to affect marmot FGM levels were included as fixed effects: current predation pressure (Blumstein et al. 2016; Monclús et al. 2011), age (Armitage 2014b, Armitage, 1998), elevation (higher or lower); (Armitage 2014a; Blumstein et al. 2004; Van Vuren and Armitage 1991), reproductive status (whether they bred that year and, for those who did, whether the sample was collected before or after the pups had been weaned; Smith et al. 2012), day of the year (Smith et al. 2012), time of sample collection (Smith et al. 2012), and June body mass (Wey et al. 2015; see Appendix A for more detailed descriptions and justifications). Lastly, our previous investigation of cumulative ELA (Ortiz‐Ross and Blumstein 2024) showed a significant interaction between our CAI and elevation, so we included this interaction in our model. The individual ID and year were then included as random effects.

We additionally fit another linear mixed model (hereafter the “full” model) in which the CAI was replaced by the adversity measures themselves (in their binary coding; Appendix B). However, we were unable to include an interaction with elevation without overfitting the model. All analyses were conducted in R (R Core Team 2023) using R packages *lme4* (Bates et al. 2015) and *lmerTest* (Kuznetsova et al. 2017). Model diagnostics were run using the *performance* package (Lüdecke et al. 2021).

## 3 Results

Our final data set consisted of 511 fecal samples for 80 unique adult females across 17 years (2004–2020). Overall, the population experienced a mean of 2.03 instances of adversity (range 0–8), with individuals at higher elevation experiencing slightly higher adversity than those at lower elevation (respective means of 2.57 and 1.18).

After accounting for potential sources of variation in metabolism and current environmental stressors, we found a significant interaction between ELA and elevation (Table 1). At lower elevation, FGMs decreased with increasing adversity; but at higher elevation, FGMs showed a slight increase with increasing adversity (Figure 2). However, none of our effect sizes were particularly large, suggesting that yellow‐bellied marmots maintain relatively stable FGMs across environmental conditions. Together, these results suggest that yellow‐bellied marmots are a resilient species that seem to maintain a tightly regulated stress response, particularly under harsh environmental conditions.

**TABLE 1:** Results from the linear mixed‐effects model showing the association between a Cumulative Adversity Index (CAI) and female adult fecal glucocorticoid metabolites (FGMs) in yellow‐bellied marmots.

Fixed effects	Estimates	CI	p
(Intercept)	0.0748	−0.5227 to 0.6723	0.806
CAI	−0.0645	−0.1340 to 0.0051	0.069
Elevation [high]	0.0095	−0.1713 to 0.1903	0.918
Predation pressure [low]	−0.043	−0.1429 to 0.0568	0.398
Lactating (pre‐wean)	**−0.1421**	**−0.2410** to **−0.0432**	**0.005**
Non‐breeding	0.0079	−0.1024 to 0.1182	0.888
Day of year	**−0.0047**	**−0.0063** to **−0.0030**	**< 0.001**
Time (radians)	**0.0679**	**0.0328** to **0.1031**	**< 0.001**
June mass	0.00004	−0.0001 to 0.0002	0.673
Age	0.0003	−0.0330 to 0.0336	0.987
CAI × elevation [high]	**0.0774**	**0.0032** to **0.1517**	**0.041**

Random effects	
σ^2^	0.14
τ_00 uid_	0.02
τ_00 year_	0.15
ICC	0.55
Marginal *R* ^2^/conditional *R* ^2^	0.073/0.583

*Note:* The data set included 511 fecal samples for 80 unique adult females across 17 years. Bold values are associated with a *p* ≤ 0.05.

**FIGURE 1:**
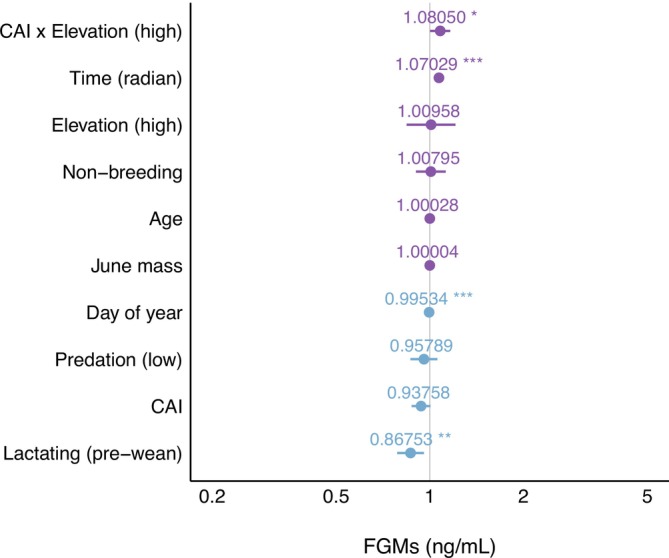
Plot of the estimated change in fecal glucocorticoid metabolite (FGM) levels for each fixed effect in our linear mixed effects model. CAI stands for cumulative adversity index, which measures the number of ELAs individuals encountered. Elevation has been categorically split into higher and lower elevations, consistent with past studies. The data set included 511 fecal samples for 80 unique adult female yellow‐bellied marmot individuals across 17 years. Random effects of year and ID were included. Asterisks denote statistical significance: *p ≤ 0.05; **p ≤ 0.01; ***p ≤ 0.001.

The covariates in our model mostly yielded expected trends based on previous studies and biological relevance (Table 1 and Figure 1). Of these, reproductive status (pre‐wean) had the largest effect size, followed by the interaction between CAI and elevation and time of day. Post‐weaning females (whose pups had emerged from their burrows) had significantly higher levels of FGMs than pre‐weaning individuals, a previously reported finding (Smith et al. 2012). A likely consequence is the increased predation risk to pups. Interestingly, here we found no strong effects of predation pressure, age, and mass, even though they have had modest effects in past studies. ELA appears to have a stronger effect and be a better predictor of FGMs. Nevertheless, the random effect of year explained substantial variation in FGMs in this species.

## 4 Discussion

**FIGURE 2:**
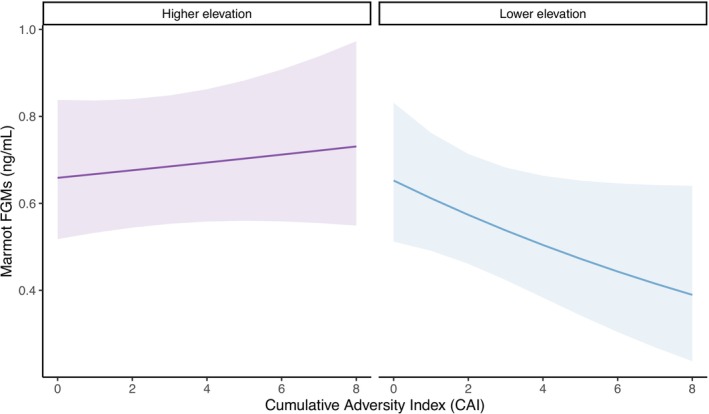
Plot of the predicted change in fecal glucocorticoid metabolite (FGM) levels in yellow‐bellied marmots in response to increasing number of early‐life adversities (measured using a cumulative adversity index or CAI) and across an elevational difference (~300–400 m, categorically and historically divided into higher and lower elevations).

While prior work in humans (Dich et al. 2015; Hammen et al., 2000; McLaughlin et al. 2010) and other free‐living vertebrates (Laubach et al. 2021; Rosenbaum et al. 2020; Vargas et al. 2016) has shown ELA may have costly effects on stress physiology later in life, we found only moderate support for this in our longitudinal investigation of wild yellow‐bellied marmots. Positive relationships between GCs and ELA are often assumed and have been found in other animals. Spotted hyenas ( *Crocuta crocuta* ) that experienced greater social connectedness in early life showed lower FGM levels, suggesting that early social connections led to a healthier stress response (Laubach et al. 2021). In Sprague–Dawley rats, social isolation in early life, more specifically during rearing, was associated with higher levels of basal corticosterone and stress reactivity in adults (Vargas et al. 2016). However, in yellow‐bellied marmots, the relationship between ELA and adult FGM levels varied depending on elevation. Higher elevation marmots exhibited little to no increase in FGMs as ELA increased, while lower elevation marmots had FGM levels that decreased with increasing ELA (Figure 2).

These results convey some support for the hypothesis that the accumulation of ELA results in an altered or dysregulated stress response (although the HPA response was not directly measured in this study). However, contrary to similar research in other animals (Laubach et al. 2021; Patterson et al. 2021; Rosenbaum et al. 2020), the trend suggested down‐regulation rather than up‐regulation under more environmentally relaxed conditions. A decrease in FGMs with increasing ELA could indicate habituation or a lower functioning stress response. Whether such low FGM levels are detrimental or beneficial remains to be determined. Importantly, these results are consistent with some human studies that have found positive associations between ELA and down‐regulation (or blunted reactivity) of the stress response (Evans and Kim 2007; Lovallo et al. 2000). However, these results could also indicate that only individuals with lower FGMs were able to survive to adulthood, an unavoidable bias in the data.

On the other hand, the lack of substantial change in FGM levels at higher elevation (although there was a slight increase in FGMs) suggests that marmots that grow up under harsher conditions may be either more resilient to adversity or chronically stressed due to their current harsh environment. Similarly, a previous study that looked at the effects of ELA on longevity showed that while annual marmot survival was lower at higher elevation, it was less susceptible to increases in ELA (Ortiz‐Ross and Blumstein 2024). Perhaps maintaining high FGM levels when living in harsh conditions helps shield individuals from some of the costs of ELA.

It must be noted that our models assumed each adversity measure was equally as important as the next (individual stressors were not weighted). While the reasons for this decision are discussed and supported elsewhere (Ortiz‐Ross and Blumstein 2024), future studies could examine this assumption in more detail. Furthermore, our sample was limited to 80 individuals for whom we had lifelong data and included only those who made it to adulthood. Given that cumulative ELA considerably reduces first‐year survival in marmots (Ortiz‐Ross and Blumstein 2024), it is likely that many individuals with high CAI values died early and did not make it into our study. However, those who survived seem to pay little additional costs in terms of stress (or metabolic) physiology.

Marmots have a highly seasonal lifestyle; each fall, they begin a 7–8‐month hibernation. Survivors emerge in spring and must obtain sufficient resources to reproduce and ensure they survive the winter hibernation. However, consecutive years are not identical; current stressors such as food abundance or predation may fluctuate, and these unpredictable fluctuations may become enhanced with climate change. It is possible that, by being adapted to natural variations in stressors and/or by undergoing drastic metabolic changes seasonally, marmots are also adapted to be resilient to ELA. Similar studies on other highly seasonal species are required to evaluate this hypothesis, which could have important implications for our understanding of how animals respond to ELA.

In addition to providing evidence for long‐term physiological consequences of ELA in marmots, our study also highlights the utility of using a CAI framework to account for multiple co‐occurring stressors and, indeed, to better understand how ELA accumulates in free‐living animals—an essential step toward understanding resilience. Indeed, our full models did not show any particular adversity measure as responsible for the significant association between the CAI and adult FGMs (Appendix B). It is therefore imperative that we consider cumulative impacts as we develop a better mechanistic understanding of resilience. Only then will we be able to develop more realistic demographic models to help us manage species in our rapidly changing world.

## Author Contributions

**Alyssa Y. Kong:** data curation (equal), formal analysis (equal), investigation (equal), writing – original draft (equal). **Xochitl Ortiz‐Ross:** conceptualization (lead), data curation (lead), formal analysis (lead), funding acquisition (supporting), investigation (lead), methodology (equal), project administration (lead), supervision (equal), validation (lead), visualization (lead), writing – original draft (equal), writing – review and editing (lead). **Daniel T. Blumstein:** conceptualization (supporting), funding acquisition (lead), investigation (equal), methodology (equal), resources (lead), supervision (lead), writing – review and editing (supporting).

## Conflicts of Interest

The authors declare no conflicts of interest.

## Data Availability

The data and code for the analyses and figures that support the findings of this study are available on OSF at: https://osf.io/mv3q8/?view_only=8ec46f4965144a119cefb7d2c0c98f7d.

## Appendix A Summary of selected covariates and reasoning for inclusion in the model

**Table:**

Covariate	Description	Reasoning for inclusion	Reference
Predation pressure	The current predator index indicates areas of high predation and areas of low predation based on a median split of observed predator frequency per colony per year	Marmots have greater FGMs in areas of high predation compared to areas with low predation	(Blumstein et al. 2016; Monclús et al. 2011)
Age	Age was determined based on birth year. Only individuals of known age were included	Two and three‐year old females are reproductively suppressed by older breeding‐age females (often their mothers), which can increase their FGMs	(Armitage 2014b, 1998)
Elevation	Valley elevation was scored either as “higher” or “lower”	At our study site, higher elevation conditions are more stressful than lower elevation ones because of a shorter growing season and (often) greater predation risk	(Armitage 2014a; Blumstein et al. 2004; Van Vuren and Armitage 1991)
Day of year	The day of marmot fecal collection	FGM values decrease as the active season progresses such that FGM values in feces collected during September are lower than those collected earlier	(Smith et al. 2012)
Time of day	Time at which the marmot was trapped and the fecal sample collected	FGM values increase throughout the day such that FGM values are greater in feces collected in the evening than in feces collected in the morning	(Smith et al. 2012)
June body mass	Marmots are weighed when trapped. We used June body mass as a measure of body condition. June mass was predicted from a linear mixed effect model	Long winters leave survivors in relatively poor body condition and must forage more to regain condition	(Wey et al. 2015)
Breeding status	Whether the female reproduced in the year that the samples were collected. If they bred, we classified the samples as pre‐weaning (or while the breeding female was lactating) and post‐weaning (or after the breeding female's pups were done lactating and emerged from their burrows)	Pregnancy increases FGM levels. Mothers with recently emerged young generally have higher FGMs than before weaning	(Smith et al. 2012)

## Appendix B Supplementary analysis—results of the ‘full’ model, a model that included each adversity measure, in its binary form, as individual covariates. Due to the complexity of this model, we were unable to include an interaction between each adversity measure and elevation to replace the significant interaction between the cumulative adversity index (CAI) and elevation.

**Table:**

Log(FGMs in ng/mL)			
Predictors	Estimates	CI	*p*
(Intercept)	−0.1838	−0.79 to 0.42	0.552
Late growing season (ELA)	0.0269	−0.13 to 0.18	0.730
Summer drought (ELA)	**−0.1585**	**−0.28** to **−0.04**	**0.008**
High early predation (ELA)	0.0655	−0.01 to 0.15	0.108
Male‐biased litter (ELA)	0.1246	−0.12 to 0.36	0.308
Large litter size (ELA)	0.1376	−0.03 to 0.31	0.114
Late weaning (ELA)	0.0001	−0.16 to 0.16	0.999
Low maternal mass (ELA)	−0.0124	−0.30 to 0.27	0.932
High maternal stress (ELA)	−0.0696	−0.20 to 0.06	0.287
Maternal loss (ELA)	0.0104	−0.07 to 0.09	0.798
Higher elevation	**0.1894**	**0.06** to **0.32**	**0.004**
Current predation	−0.0481	−0.15 to 0.05	0.343
Lactating (pre‐wean)	**−0.1434**	**−0.24** to **−0.04**	**0.005**
Non‐breeding	0.0018	−0.11 to 0.11	0.975
Day of year	**−0.0046**	**−0.01** to **−0.00**	**< 0.001**
Time of day (radians)	**0.0657**	**0.03** to **0.10**	**< 0.001**
June mass (g)	0.0001	−0.00 to 0.00	0.295
Age	0.0003	−0.03 to 0.04	0.987
Random effects			
σ^2^	0.14		
τ_00 uid_	0.01		
τ_00 year_	0.16		
ICC	0.55		
Marginal *R* ^2^/conditional *R* ^2^	0.093/0.593		

*Note:* The data set included 511 fecal samples from 80 unique adult females over 17 years.
